# Robotic-assisted vs. open and laparoscopic Kasai portoenterostomy for biliary atresia: a systematic review and meta-analysis

**DOI:** 10.3389/fped.2026.1916128

**Published:** 2026-08-17

**Authors:** Yuhan He, Weiwei Liu

**Affiliations:** 1 School of Medical and Life Sciences, Chengdu University of Traditional Chinese Medicine, Chengdu, China; 2 School of Basic Medical Sciences, Chengdu University of Traditional Chinese Medicine, Chengdu, China

**Keywords:** biliary atresia, laparoscopic surgery, meta-analysis, portoenterostomy, hepatic, robotic-assisted surgery

## Abstract

**Background:**

Biliary atresia is a progressive neonatal cholangiopathy that requires timely Kasai portoenterostomy to restore bile drainage and improve native liver survival. Robotic-assisted Kasai portoenterostomy (RAKPE) has recently emerged as a potential minimally invasive alternative to open Kasai portoenterostomy (OKPE) and laparoscopic Kasai portoenterostomy (LKPE). However, its comparative efficacy and safety remain uncertain.

**Methods:**

We searched PubMed, EMBASE, and the Cochrane Library for comparative studies published from January 1, 2000, to May 20, 2026. Eligible studies included children with biliary atresia who underwent RAKPE and were compared with patients treated by OKPE and/or LKPE. The primary outcomes were 6-month jaundice clearance and 1-year native liver survival. Odds ratios (ORs) were calculated for dichotomous outcomes, and mean differences (MDs) were calculated for continuous outcomes. RAKPE was analyzed separately against OKPE and LKPE.

**Results:**

Five retrospective comparative studies were included. RAKPE showed short-term clinical outcomes that were generally comparable to those of OKPE and LKPE. The pooled estimates suggested a possible tendency toward higher jaundice clearance after RAKPE, but the differences were not statistically significant. Sensitivity analyses indicated that RAKPE might be associated with less intraoperative blood loss and shorter hospital stay in selected comparisons. These perioperative findings were influenced by individual studies and should therefore be interpreted as preliminary.

**Conclusion:**

Current comparative evidence suggests that RAKPE can achieve jaundice clearance, native liver survival, and postoperative cholangitis outcomes similar to those of OKPE and LKPE in children with biliary atresia. The robotic approach may have advantages in selected perioperative outcomes, but longer operative time remains a consistent drawback. Because the available evidence is based on a small number of retrospective studies, multicenter prospective studies with standardized outcome definitions and longer follow-up are needed before the role of RAKPE can be defined more firmly.

**Systematic Review Registration:**

https://www.crd.york.ac.uk/PROSPERO/view/CRD420261401089, PROSPERO CRD420261401089.

## Introduction

Biliary atresia (BA) is a severe neonatal cholangiopathy characterized by progressive inflammatory and fibrotic obstruction of the intrahepatic and extrahepatic bile ducts. Without timely treatment, BA may rapidly progress to biliary cirrhosis, liver failure, and death in early childhood (1). Kasai portoenterostomy (KPE) remains the first-line surgical treatment for BA. The operation aims to restore bile drainage by removing the fibrotic biliary remnant at the porta hepatis and constructing a portoenterostomy (2). Although KPE has greatly improved the prognosis of BA, long-term outcomes are still variable, and many children eventually require liver transplantation (1, 3). In clinical practice,jaundice clearance is often used to assess early surgical success, whereas native liver survival (NLS) provides a more meaningful measure of longer-term benefit (4, 5).

OKPE has long been regarded as the standard surgical approach for BA. With the development of minimally invasive surgery, LKPE was introduced in the early twenty-first century (6). LKPE may reduce surgical trauma, improve cosmetic outcomes, and accelerate postoperative recovery. However, the value of LKPE remains debated because KPE is technically demanding in infants. Precise dissection of the fibrous cone at the porta hepatis and construction of an adequate portoenterostomy are difficult to perform in a small operative field (7, 8). A previous meta-analysis comparing LKPE with OKPE found no clear superiority in jaundice clearance, postoperative cholangitis, or NLS, although operative time and intraoperative blood loss differed between the two approaches (9).

RAKPE has emerged as a potential alternative surgical approach for BA. Compared with conventional laparoscopy, robotic systems provide three-dimensional visualization, tremor filtration, motion scaling, and wristed instruments, which may facilitate precise hilar dissection and intracorporeal suturing (7, 8). These technical features may theoretically help overcome some limitations of LKPE, particularly in fibrous cone dissection and portoenterostomy anastomosis. Early reports suggested that RAKPE was technically feasible and could achieve acceptable short-term outcomes, but the available evidence was initially limited to small case series without eligible control groups (8). Therefore, whether RAKPE provides comparable or superior outcomes to OKPE or LKPE remains uncertain.

Recently, comparative studies evaluating RAKPE against OKPE or LKPE have gradually increased (10–14). A previous network meta-analysis comparing different KPE techniques suggested that RAKPE might rank favorably for postoperative clearance of jaundice; however, no statistically significant differences were detected among RAKPE, LKPE, and OKPE (15). Moreover, that analysis combined different surgical comparisons within a network framework and included limited robotic data. Given the increasing number of direct comparative studies, an updated pairwise meta-analysis focusing specifically on RAKPE is needed. In particular, RAKPE should be compared separately with OKPE and LKPE to avoid inappropriate pooling of different control approaches and to better clarify its relative efficacy and safety.

Therefore, this systematic review and meta-analysis aimed to evaluate the short-term clinical efficacy and perioperative safety of RAKPE compared with OKPE and LKPE in children with BA. The main outcomes included 6-month jaundice clearance and 1-year NLS, while secondary outcomes included postoperative cholangitis, liver transplantation, operative time, intraoperative blood loss, time to oral feeding, and length of hospital stay. By synthesizing the currently available comparative evidence, this study sought to clarify whether RAKPE can be considered a feasible and safe alternative to conventional KPE approaches.

## Methods

### Protocol and registration

This systematic review follows PRISMA (Preferred Report-ing Items for Systematic Reviews and Meta-Analyses) guidelines (16, 17). The study protocol was registered on PROSPERO (ID: CRD420261401089).

### Search strategy

A comprehensive literature search was conducted in PubMed, EMBASE, and the Cochrane Library. Studies published between January 1, 2000, and May 20, 2026, were considered eligible. The search was restricted to studies published from 2000 onward because laparoscopic and robotic surgical techniques were introduced and increasingly applied in clinical practice after this period (6–8). MeSH terms, including “Biliary Atresia” and “Portoenterostomy, Hepatic”, were used, and free-text terms were derived accordingly. The complete search strategies are provided in the Supplementary Data Sheet 1.

### Inclusion and exclusion criteria

Studies were considered eligible if they directly compared RAKPE with OKPE and/or LKPE in infants or children diagnosed with BA. Eligible studies had to report at least one extractable clinical or perioperative outcome, including jaundice clearance, NLS, postoperative cholangitis, complications, liver transplantation, operative time, intraoperative blood loss, time to oral feeding, or length of hospital stay. For quantitative synthesis, studies also needed to provide sufficient numerical data, such as event numbers for dichotomous outcomes or means and standard deviations for continuous outcomes.

Studies were excluded if they were unrelated to BA, KPE, or robotic-assisted surgery. Reviews, meta-analyses, letters, commentaries, editorials, expert opinions, and conference abstracts without sufficient original data were also excluded. Single-arm studies or case series without an OKPE or LKPE comparator were not eligible. Studies lacking extractable data for the prespecified outcomes were excluded. When overlapping patient populations were suspected, the most complete or most recent report was retained to avoid duplicate inclusion. Animal studies, experimental studies, and purely technical reports without eligible comparative clinical data were excluded.

### Outcome definitions

The primary outcomes were 6-month jaundice clearance (JC) and 1-year native liver survival (NLS). Six-month JC was defined as a serum total bilirubin concentration of <20 μmol/L at or within 6 months after Kasai portoenterostomy. When an original study used a threshold of ≤20 μmol/L rather than <20 μmol/L, the two definitions were considered clinically equivalent, and this minor discrepancy was recorded during data extraction. One-year NLS was defined as survival with the native liver at 1 year after surgery. Death or liver transplantation before the 1-year follow-up point was classified as failure of NLS.

Secondary outcomes included postoperative cholangitis, overall postoperative complications, liver transplantation, operative time, intraoperative blood loss, time to oral feeding, and length of hospital stay. Postoperative cholangitis was defined according to the diagnostic criteria reported in each original study. Where explicitly stated, the diagnosis was generally based on compatible clinical features, such as fever, recurrent jaundice, or acholic stools, together with inflammatory or liver biochemical abnormalities. Because the exact clinical and laboratory criteria were not uniform across the included studies, the study-specific definitions were extracted and the discrepancies were considered when interpreting heterogeneity.

Overall postoperative complications were defined as any surgery-related adverse event reported by the original investigators. The included studies did not consistently apply a standardized severity-grading system, such as the Clavien–Dindo classification. Therefore, complications were analyzed according to the definitions used in the original studies, and complication severity was not pooled. Liver transplantation was defined as transplantation performed during the follow-up period reported by each study. Operative time, intraoperative blood loss, time to oral feeding, and length of hospital stay were extracted according to the definitions and units reported in the original studies.

### Data extraction and quality assessment

Two reviewers independently extracted data according to the predefined criteria. Extracted information included study characteristics, patient baseline data, surgical approach, clinical outcomes, outcome definitions, serum bilirubin cut-off values, assessment time points, diagnostic criteria for postoperative cholangitis, and complication-grading systems. Any discrepancies in outcome definitions among the included studies were recorded during data extraction. Disagreements were resolved through discussion and consensus between the two reviewers. Methodological quality was assessed independently by two reviewers using the Newcastle–Ottawa Scale (NOS) (18). The NOS evaluates nonrandomized studies across the domains of selection, comparability, and outcome assessment, with a maximum score of nine stars. Any disagreement in quality assessment was resolved by consensus.

### Data synthesis and statistical analysis

Meta-analysis was performed when at least two studies reported the same outcome using sufficiently comparable definitions and follow-up time points. Minor differences in the definition of JC, specifically the use of <20 μmol/L or ≤20 μmol/L as the serum total bilirubin threshold, were considered clinically equivalent. Outcomes with substantially different definitions, diagnostic criteria, or assessment time points were not quantitatively pooled and were summarized narratively where appropriate.Because OKPE and LKPE differ in surgical exposure and technical characteristics, RAKPE was compared separately with each approach. Dichotomous outcomes, including jaundice clearance, NLS, postoperative cholangitis, complications, and liver transplantation, were pooled using ORs with 95% confidence intervals (CIs). Continuous outcomes, including operative time, intraoperative blood loss, time to oral feeding, and length of hospital stay, were pooled using MDs with 95% CIs.

Heterogeneity was assessed using the Chi-square test and the I² statistic. An I² value greater than 50% was considered to suggest substantial heterogeneity (19). Random-effects models were used as the primary models because clinical and methodological differences were expected among the included studies. Sensitivity analyses were conducted by changing the statistical model or excluding studies that appeared to contribute to heterogeneity. Publication bias was not assessed with funnel plots or statistical tests because fewer than 10 studies were included in each comparison. All analyses were performed using Review Manager 5.4.

## Results

### Study selection

**Figure 1:**
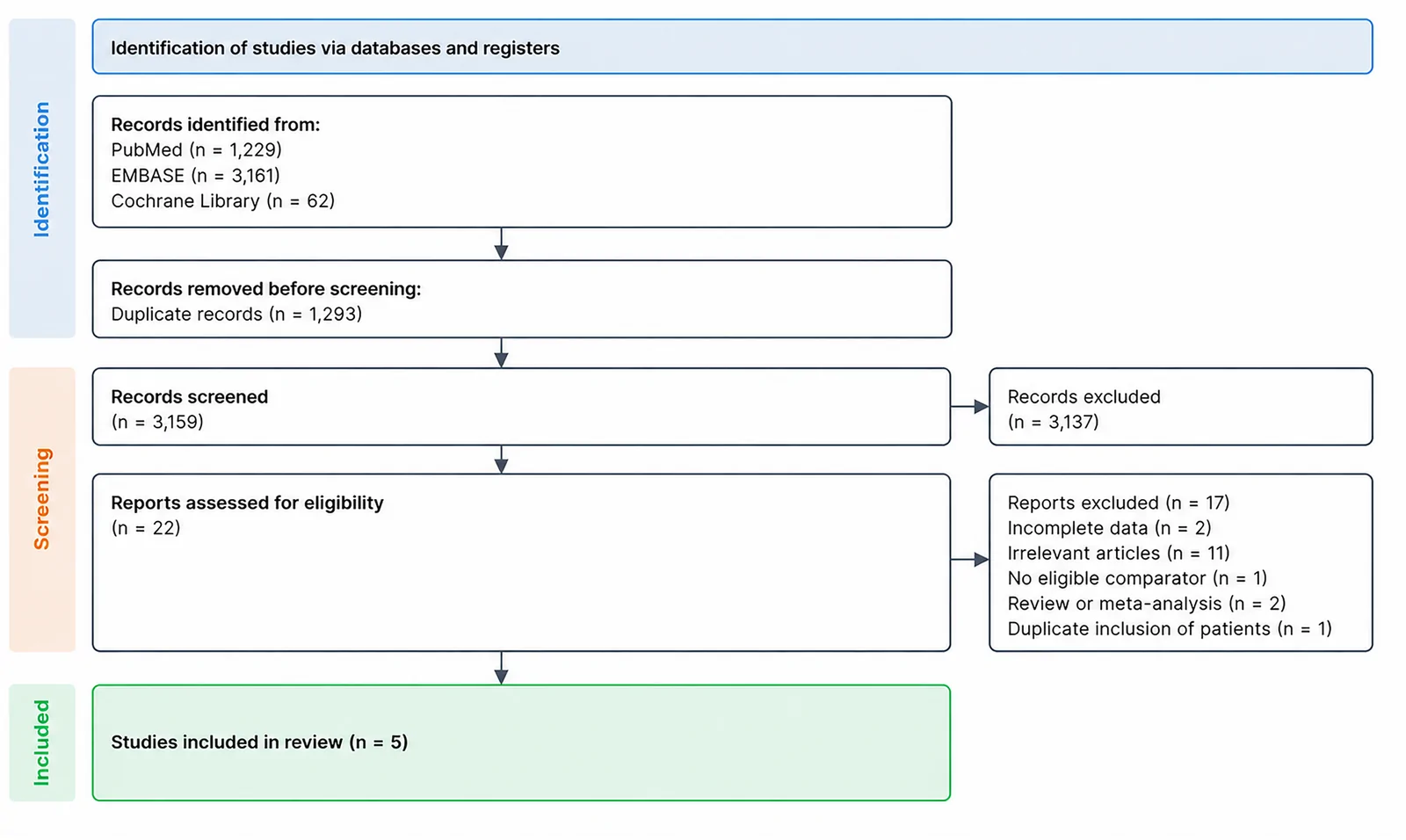
PRISMA flow diagram of the study selection process.

A total of 4,452 records were identified through database searches, including 1,229 from PubMed, 3,161 from EMBASE, and 62 from the Cochrane Library. After removing 1,293 duplicate records, 3,159 records were screened by title and abstract, of which 3,137 were excluded. The full texts of 22 reports were assessed for eligibility, and 17 were excluded because of incomplete data (*n* = 2), irrelevant articles (*n* = 11), no eligible comparator (*n* = 1), review or meta-analysis (*n* = 2), or duplicate inclusion of patients (*n* = 1). Finally, five studies were included in the systematic review and meta-analysis (10–14). The study selection process is presented in Figure 1.

#### Baseline characteristics

The characteristics of the included studies are summarized in Table 1. Five comparative studies published between 2024 and 2026 were included, with KPE performed between 2015 and 2024. All studies were conducted in China and used a retrospective cohort design. To avoid duplicate counting, studies with overlapping patient populations were carefully assessed, and only the most complete or most recent report was retained. RAKPE was compared separately with OKPE and LKPE, and data from multi-arm studies were entered only into the relevant pairwise comparisons. Three studies compared RAKPE with OKPE, one compared RAKPE with LKPE, and one three-arm study reported data for RAKPE, OKPE, and LKPE. In total, 374 patients undergoing KPE were included, with 135 in the RAKPE group, 129 in the OKPE group, and 110 in the LKPE group. The mean or median age at surgery ranged from approximately 53.5–66.1 days. Reported baseline characteristics included sex distribution, body weight, surgical approach, sample size, age at operation, and perioperative variables. The outcome measures reported in each included study are summarized in Table 2.

**Table 1:** The baseline characteristics of the included studies.

No.	Study	Study period	Institution	Study design	Sex (M:F)	Approach	*n*	Age at operation (days)
1	Guo et al. (10), 2025	2018.01–2022.12	Union Hospital, Tongji Medical College, Huazhong University of Science and Technology, Wuhan	Retrospective cohort	16:20	RAKPE	36	59.72 ± 12.60
					17:21	OKPE	38	63.18 ± 14.10
2	Zheng et al. (11), 2024	2022.01–2023.12	Children Hospital of Guizhou Province/Affiliated Hospital of Zunyi Medical University	Retrospective cohort; two centers	7:6	RAKPE	13	55.20 ± 22.10
					8:10	OKPE	18	60.10 ± 17.30
3	Xing et al. (14), 2024	2018.12–2021.12	Seventh Medical Center of PLA General Hospital, Beijing	Retrospective cohort	3:6	RAKPE	9	66.11 ± 15.04
					30:22	OKPE	52	63.98 ± 15.73
4	Jiang et al. (12), 2026	2015.01–2024.12	Children's Hospital of Soochow University, Suzhou	Retrospective cohort	5:6	RAKPE	11	60 ± 10
					9:12	OKPE	21	61 ± 10
					7:11	LKPE	18	57 ± 11
5	Zhang et al. (13), 2025	2018.10–2023.06	Multicenter: Union Hospital, Zunyi/Guizhou, and participating center(s)	Multicenter retrospective cohort	26:40	RAKPE	66	53.50 (45.75–60.50)
					41:51	LKPE	92	55.00 (46.25–63.00)

RAKPE, robotic-assisted Kasai portoenterostomy; OKPE, open Kasai portoenterostomy; LKPE, laparoscopic Kasai portoenterostomy.

**Table 2:** Outcome measures reported in the included studies.

(A) Clinical outcomes					
Study	Comparison	6-month JC	1-year NLS	Postoperative cholangitis	Liver transplantation
Guo et al. (10), 2025	RAKPE vs. OKPE	✓		✓	
Zheng et al. (11), 2024	RAKPE vs. OKPE	✓		✓	✓
Xing et al. (14), 2024	RAKPE vs. OKPE				
Jiang et al. (12), 2026	RAKPE vs. OKPE; RAKPE vs. LKPE	✓	✓	✓	
Zhang et al. (13), 2025	RAKPE vs. LKPE	✓	✓	✓	✓

(B) Perioperative outcomes					
Study	Comparison	Operative time	Blood loss	Hospital stay	Time to oral feeding
Guo et al. (10), 2025	RAKPE vs. OKPE	✓	✓	✓	
Zheng et al. (11), 2024	RAKPE vs. OKPE	✓	✓	✓	✓
Xing et al. (14), 2024	RAKPE vs. OKPE	✓	✓	✓	✓
Jiang et al. (12), 2026	RAKPE vs. OKPE; RAKPE vs. LKPE	✓	✓	✓	✓
Zhang et al. (13), 2025	RAKPE vs. LKPE	✓	✓	✓	

JC, jaundice clearance; NLS, native liver survival; RAKPE, robotic-assisted Kasai portoenterostomy; OKPE, open Kasai portoenterostomy; LKPE, laparoscopic Kasai portoenterostomy. A check mark indicates that extractable comparative data were available for that outcome. Guo et al. reported SNL during follow-up but did not clearly define it as a 1-year NLS endpoint.

### Methodological quality assessment

The methodological quality of the included studies was assessed using the Newcastle–Ottawa Scale. The total NOS scores ranged from 8 to 9 points. Guo et al. (10), Zheng et al. (11), and Xing et al. (14) received 8 points, whereas Jiang et al. (12) and Zhang et al. (13) received 9 points. All five studies were therefore considered to be of relatively high methodological quality according to the NOS criteria. However, because all included studies had retrospective cohort designs, potential selection bias and residual confounding could not be completely excluded. The detailed domain-level assessments are presented in Table 3.

**Table 3:** Methodological quality assessment of the included studies using the Newcastle-Ottawa scale.

Study	Selection (maximum 4)	Comparability (maximum 2)	Outcome (maximum 3)	Total score (maximum 9)	Quality
Guo et al. (10), 2025	4/4	2/2	2/3	8	High
Zheng et al. (11), 2024	4/4	1/2	3/3	8	High
Xing et al. (14), 2024	4/4	1/2	3/3	8	High
Jiang et al. (12), 2026	4/4	2/2	3/3	9	High
Zhang et al. (13), 2025	4/4	2/2	3/3	9	High

NOS, Newcastle-Ottawa Scale. Scores are presented as points awarded/maximum available points. Studies with a total score of 7–9 were considered high quality.

### Meta-analysis

Separate pairwise meta-analyses were performed for RAKPE versus OKPE and RAKPE versus LKPE. To distinguish the clinical importance of the outcomes and avoid repetitive reporting, the findings were grouped into core efficacy outcomes, surgical morbidity, and postoperative recovery.

### Core efficacy outcomes

**Figure 2:**
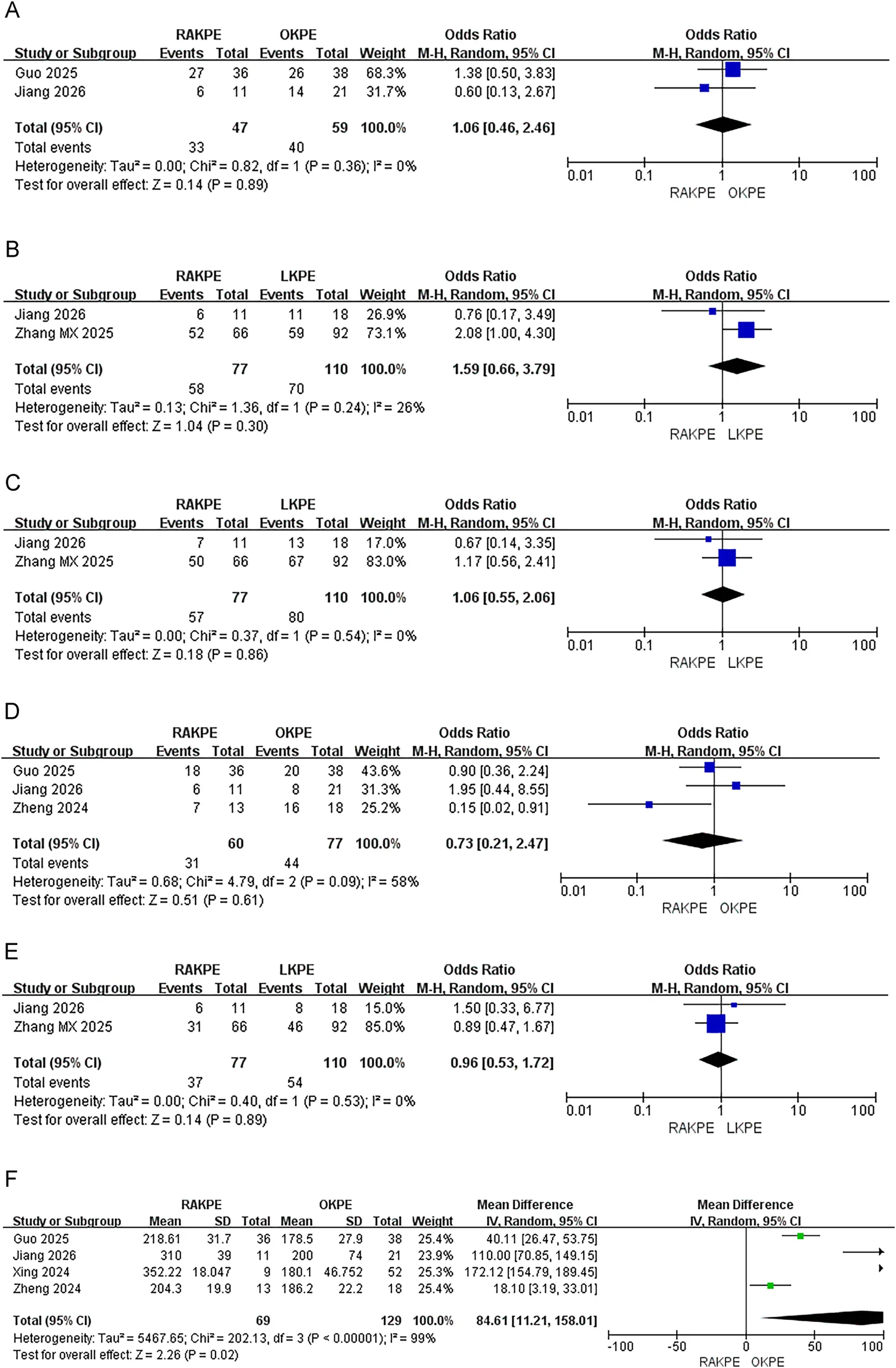

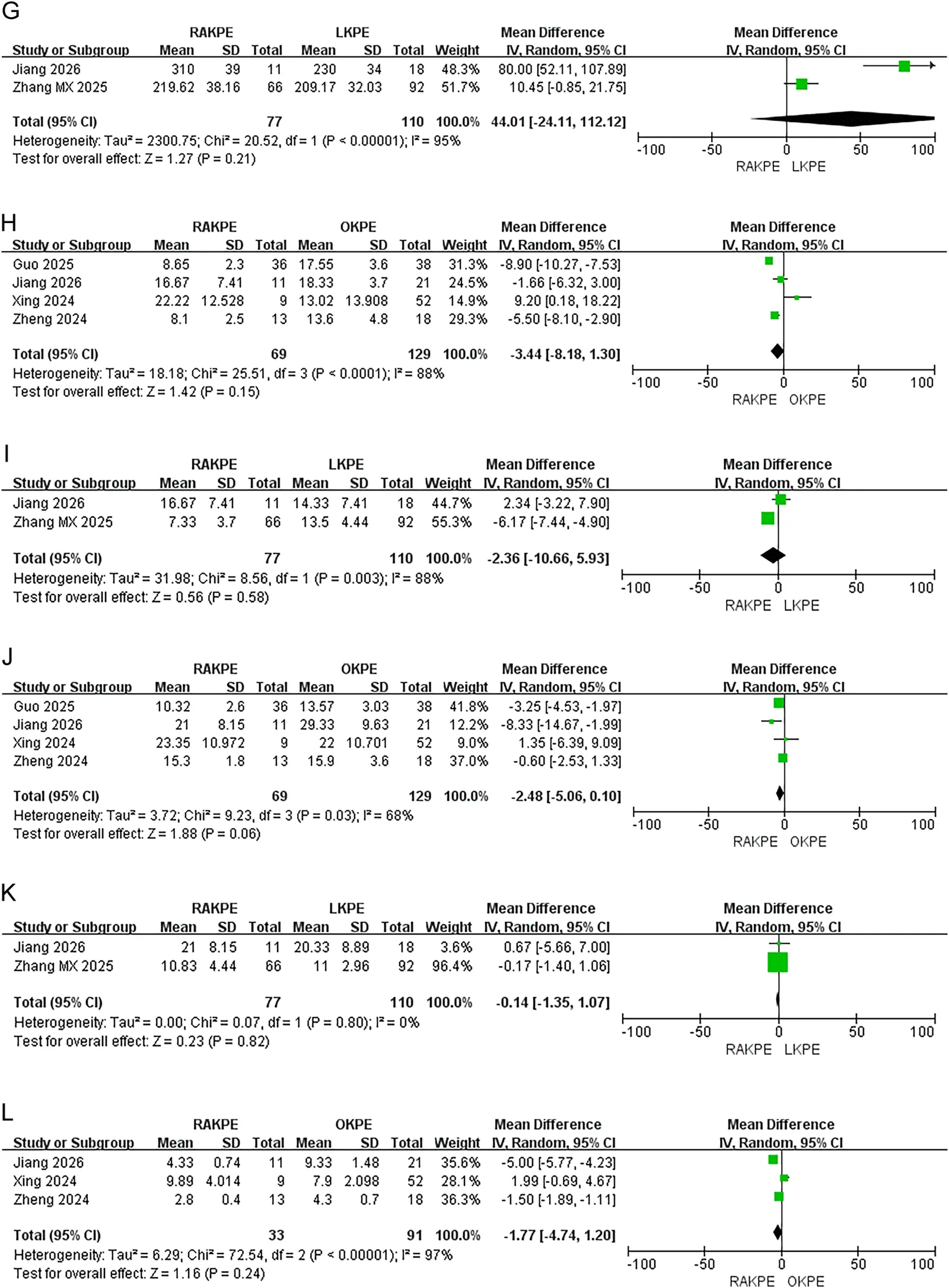
Forest plots of RAKPE vs. OKPE and RAKPE vs. LKPE. **(A)** 6-month JC: RAKPE vs. OKPE. **(B)** 6-month JC: RAKPE vs. LKPE. **(C)** 1-year NLS: RAKPE vs. LKPE. **(D)** Cholangitis: RAKPE vs. OKPE. **(E)** Cholangitis: RAKPE vs. LKPE. **(F)** Operative time: RAKPE vs. OKPE. **(G)** Operative time: RAKPE vs. LKPE. **(H)** Blood loss: RAKPE vs. OKPE. **(I)** Blood loss: RAKPE vs. LKPE. **(J)** Hospital stay: RAKPE vs. OKPE. **(K)** Hospital stay: RAKPE vs. LKPE. **(L)** Time to oral feeding: RAKPE vs. OKPE.

Six-month jaundice clearance was comparable between RAKPE and both conventional approaches. No significant difference was found between RAKPE and OKPE (OR 1.06, 95% CI 0.46–2.46; *P* = 0.89; I² = 0%) or between RAKPE and LKPE (OR 1.59, 95% CI 0.66–3.79; *P* = 0.30; I² = 26%) (Figures 2A,B). Although the pooled estimate for the RAKPE-versus-LKPE comparison numerically favored RAKPE, the confidence interval crossed the line of no effect. One-year native liver survival was reported only for the RAKPE-versus-LKPE comparison and was similar between the two approaches (OR 1.06, 95% CI 0.55–2.06; *P* = 0.86; I² = 0%) (Figure 2C). Overall, RAKPE did not demonstrate a significant advantage over OKPE or LKPE in the primary efficacy outcomes.

### Surgical morbidity

The incidence of postoperative cholangitis did not differ significantly between RAKPE and OKPE (OR 0.73, 95% CI 0.21–2.47; *P* = 0.61; I² = 58%) or between RAKPE and LKPE (OR 0.96, 95% CI 0.53–1.72; *P* = 0.89; I² = 0%) (Figures 2D,E). Moderate heterogeneity was observed in the RAKPE-versus-OKPE analysis, whereas the RAKPE-versus-LKPE result was consistent across studies.

Operative time was the main perioperative disadvantage of RAKPE. Compared with OKPE, RAKPE was associated with a significantly longer operative time (MD 84.61 min, 95% CI 11.21–158.01; *P* = 0.02; I² = 99%) (Figure 2F). Operative time was also numerically longer for RAKPE than for LKPE, although the difference was not statistically significant (MD 44.01 min, 95% CI −24.11–112.12; *P* = 0.21; I² = 95%) (Figure 2G). Both analyses showed substantial heterogeneity.

### Postoperative recovery

No significant differences in intraoperative blood loss were found between RAKPE and OKPE (MD −3.44 mL, 95% CI −8.18–1.30; *P* = 0.15; I² = 88%) or between RAKPE and LKPE (MD −2.36 mL, 95% CI −10.66–5.93; *P* = 0.58; I² = 88%) (Figures 2H,I). The pooled estimates were directionally favorable to RAKPE but were accompanied by substantial heterogeneity.

Length of hospital stay was not significantly different between RAKPE and either comparator. A borderline trend toward a shorter stay was observed for RAKPE versus OKPE (MD −2.48 days, 95% CI −5.06–0.10; *P* = 0.06; I² = 68%), whereas the RAKPE-versus-LKPE comparison showed almost no difference (MD −0.14 days, 95% CI −1.35–1.07; *P* = 0.82; I² = 0%) (Figures 2J,K). Time to oral feeding was available only for the RAKPE-versus-OKPE comparison and did not differ significantly between the groups (MD −1.77 days, 95% CI −4.74–1.20; *P* = 0.24; I² = 97%) (Figure 2L). Thus, the primary analyses did not establish a consistent postoperative recovery advantage for RAKPE, and several estimates were affected by substantial between-study heterogeneity.

### Sensitivity analyses

Sensitivity analyses were performed for outcomes with potential heterogeneity or influential studies. For 6-month JC between RAKPE and LKPE, the result remained non-significant when RR was used instead of OR (RR 1.20, 95% CI 0.99–1.45; *P* = 0.06; I² = 0%) and when a fixed-effect model was applied (OR 1.73, 95% CI 0.90–3.31; *P* = 0.10; I² = 26%). These results suggested a stable but non-significant trend favoring RAKPE.

Regarding postoperative cholangitis in the RAKPE versus OKPE comparison, exclusion of Zheng et al. (11) reduced heterogeneity from 58% to 0%, while the pooled result remained non-significant (OR 1.11, 95% CI 0.51–2.42; *P* = 0.79). This suggested that the study by Zheng et al. (11) was the main source of heterogeneity but did not affect the overall conclusion.

To explore the influence of individual studies on continuous outcomes, the study by Xing et al. (14) was excluded because of its small sample size and possible early learning-curve effect. After exclusion, RAKPE remained associated with longer operative time (MD 50.30 min, 95% CI 15.80–84.80; *P* = 0.004), while blood loss (MD −5.87 mL, 95% CI −9.62 to −2.13; *P* = 0.002) and hospital stay (MD −2.88 days, 95% CI −5.65 to −0.11; *P* = 0.04) became significantly lower in the RAKPE group. Time to oral feeding still showed no significant difference (MD −3.23 days, 95% CI −6.66–0.20; *P* = 0.06).

### Publication bias

Publication bias was not formally assessed using funnel plots or statistical tests because fewer than 10 studies were included in each meta-analysis. Given the limited number of available studies, the potential influence of publication bias was considered qualitatively when interpreting the results.

## Discussion

In this systematic review and meta-analysis, we summarized the currently available comparative evidence on RAKPE versus OKPE and LKPE in patients with BA. Five retrospective comparative studies were included. Overall, RAKPE showed comparable short-term clinical efficacy to OKPE and LKPE in terms of 6-month JC, 1-year NLS, and postoperative cholangitis. However, RAKPE was associated with a significantly longer operative time than OKPE. For perioperative recovery outcomes, including intraoperative blood loss, hospital stay, and time to oral feeding, the pooled estimates tended to favor RAKPE, but most differences were not statistically significant in the primary analysis and were influenced by individual studies.

Viewed together, the principal outcomes give no indication that RAKPE compromises early bile drainage or short-term NLS compared with conventional Kasai procedures. These two endpoints capture different stages of treatment response: JC reflects whether bile drainage has been restored soon after surgery, whereas NLS shows whether that early benefit is maintained over time. This interpretation is consistent with previous evidence linking successful bile flow after KPE to longer NLS, while persistent jaundice is often associated with progressive liver fibrosis and eventual liver transplantation (1, 4). In our pooled analyses, the 6-month JC rate was similar between RAKPE and the two comparator approaches. The RAKPE versus LKPE comparison showed a numerical trend toward a higher clearance rate, but the confidence interval was wide and crossed the line of no effect, which makes this finding more of a possible signal than evidence of a true advantage.

This pattern also fits with what has been reported for other Kasai techniques. An earlier meta-analysis comparing LKPE with OKPE did not show a clear advantage of LKPE in NLS or postoperative cholangitis, although LKPE was associated with longer operative time and less intraoperative blood loss (9). This background is important because it suggests that a minimally invasive approach does not necessarily lead to better liver-related outcomes. A recent network meta-analysis including open, laparoscopic, and robotic-assisted KPE reported a favorable ranking for RAKPE in JC, but the differences among the three surgical techniques were not statistically significant (15). Our study addressed the question differently by focusing specifically on RAKPE and by comparing it separately with OKPE and LKPE. This avoids treating OKPE and LKPE as a single non-robotic control group and reduces the risk of double counting data from multi-arm studies.

The potential technical advantages of RAKPE may explain the favorable direction observed for some perioperative outcomes. The robotic platform provides three-dimensional visualization, tremor filtration, motion scaling, and wristed instruments, which may facilitate precise dissection around the porta hepatis and intracorporeal suturing during portoenterostomy (7, 20). These advantages are particularly relevant for Kasai surgery, where accurate excision of the fibrous cone and construction of a wide portoenterostomy are technically demanding. In the included studies, RAKPE tended to be associated with less blood loss and shorter hospital stay, especially in sensitivity analyses excluding a small early-experience study. These perioperative findings should therefore be viewed as suggestive rather than conclusive. The primary pooled analyses were not uniformly significant, and the estimates for several continuous outcomes varied considerably across studies.

The most consistent perioperative disadvantage of RAKPE was longer operative time compared with OKPE. This finding is clinically plausible. Robotic surgery requires additional time for patient positioning, trocar placement, docking, and instrument exchange. In infants with BA, the narrow operative field and small abdominal cavity further increase technical difficulty. Moreover, the learning curve of robotic Kasai surgery is likely to be important. Early technical reports highlighted limitations of robotic portoenterostomy in infants, including large instrument size, lack of tactile feedback, and reduced maneuverability in the porta hepatis (7, 20). The study by Xing et al. (14) represented early single-center robotic experience with a very small RAKPE sample, which may partly explain the greater heterogeneity in operative time and recovery outcomes. After excluding this study in the sensitivity analysis, RAKPE still required a longer operative time than OKPE, but the magnitude of difference decreased, suggesting that operative time may improve with increasing experience.

Postoperative cholangitis remains one of the most important complications after KPE and is associated with worse bile drainage and poorer native liver outcomes (4, 21, 22). In our analysis, RAKPE did not significantly reduce postoperative cholangitis compared with either OKPE or LKPE. For the RAKPE versus OKPE comparison, moderate heterogeneity was observed in the primary analysis, and exclusion of Zheng et al. (11) reduced the I² to 0% without changing the non-significant result. This pattern points to differences in follow-up and clinical practice—such as antibiotic prophylaxis, diagnostic criteria, and postoperative management—as likely contributors to the variation between studies. The evidence therefore supports RAKPE as an option for centers with established robotic expertise, not as a procedure already shown to outperform OKPE or LKPE. Choice of approach should take account of the surgeon's experience, institutional resources, the child's condition and age, and the center's ability to provide postoperative care. These operative considerations form only part of the prognosis: age at KPE, fibrosis, anatomical subtype, cholangitis, and long-term follow-up also affect native liver outcomes (4, 5, 21–23). A recent meta-analysis found better NLS when KPE was performed early, particularly before 30 days of age (5). Timely diagnosis and referral, together with consistent postoperative care, therefore remain priorities regardless of operative access (5, 15, 24).

The certainty of these findings is limited mainly by the evidence currently available. Although all included studies received relatively high NOS scores, these ratings should not be interpreted as indicating an absence of bias. The NOS primarily assesses the reporting and methodological characteristics of nonrandomized studies, whereas the retrospective design of the included studies still leaves the findings susceptible to selection bias, residual confounding, and confounding by indication. Therefore, the methodological quality scores do not overcome the inherent limitations of the available observational evidence. The small number of studies and the limited size of the RAKPE groups also reduced the precision of several pooled estimates. An additional limitation is the geographical concentration of the available evidence. All five included studies were conducted exclusively in China, predominantly at high-volume tertiary referral centers. Surgical volumes, perioperative protocols, antibiotic prophylaxis strategies, and patient referral pathways in these centers may differ substantially from those in Western countries or other healthcare systems. Differences in patient demographics, levels of surgical expertise, and postoperative care models may also influence clinical outcomes. This geographical homogeneity limits the external validity of the present findings. Therefore, the pooled results should be interpreted cautiously and should not be overgeneralized to centers with different patient populations, surgical experience, or postoperative management practices.Some outcomes were not defined or reported consistently, particularly cholangitis, time to oral feeding, and other measures of postoperative recovery. Evidence for longer-term endpoints was even more limited; 2-year and 5-year NLS and liver transplantation could not be synthesized adequately. Formal assessment of publication bias was also not appropriate because each comparison included fewer than 10 studies, so a small-study effect cannot be ruled out.

On the basis of the present comparative data, RAKPE appears to provide short-term outcomes similar to those of OKPE and LKPE and may be considered a feasible surgical alternative in appropriately experienced centers. The evidence does not, however, establish a clear advantage for the robotic approach. Longer operative time remains its most consistent drawback, while the apparent reductions in blood loss and hospital stay were less stable and need confirmation. Larger prospective studies across multiple centers, using consistent outcome definitions and longer follow-up, are needed to determine whether the technical features of RAKPE produce reproducible clinical benefits in children with BA.

## Conclusion

In the current literature, RAKPE, LKPE, and OKPE achieve broadly similar short-term clinical results. The main trade-off appears to be a longer operative time for RAKPE without clear evidence of improved JC, NLS, or cholangitis outcomes. Based on the available data, RAKPE can be considered a reasonable option in experienced centers, but there is insufficient evidence to regard it as the preferred approach. Larger comparative studies with standardized outcome reporting and longer follow-up will be important to determine whether the technical advantages of robotics translate into meaningful clinical benefit.

## Data Availability

The original contributions presented in the study are included in the article/Supplementary Material, further inquiries can be directed to the corresponding author.
