# Supplementary material for: Robotic-assisted vs. open and laparoscopic Kasai portoenterostomy for biliary atresia: a systematic review and meta-analysis
Source: Front Pediatr. 2026 Aug 17;14:1916128. doi: 10.3389/fped.2026.1916128 (PMC13527007; doi:10.3389/fped.2026.1916128)
Supplement: Supplementary file 1 [file Datasheet1.docx]

## PubMed:

| 1 | ((((((Portoenterostomy, Hepatic[MeSH Terms]) OR (Hepatoportoenterostomy[Title/Abstract])) OR (Hepatoportoenterostomies[Title/Abstract])) OR (Kasai Procedure[Title/Abstract])) OR (Hepatic Portoenterostomy[Title/Abstract])) OR (Hepatic Portoenterostomies[Title/Abstract])) OR (Portoenterostomies, Hepatic[Title/Abstract]) |
| --- | --- |
| 2 | (((((((((Biliary Atresia[MeSH Terms]) OR (Atresia, Biliary[Title/Abstract])) OR (Intrahepatic Biliary Atresia[Title/Abstract])) OR (Atresia, Intrahepatic Biliary[Title/Abstract])) OR (Biliary Atresia, Intrahepatic[Title/Abstract])) OR (Biliary Atresia, Extrahepatic[Title/Abstract])) OR (Atresia, Extrahepatic Biliary[Title/Abstract])) OR (Extrahepatic Biliary Atresia[Title/Abstract])) OR (Idiopathic Extrahepatic Biliary Atresia[Title/Abstract])) OR (Familial Extrahepatic Biliary Atresia[Title/Abstract]) |
| 3 | (((((((Portoenterostomy, Hepatic[MeSH Terms]) OR (Hepatoportoenterostomy[Title/Abstract])) OR (Hepatoportoenterostomies[Title/Abstract])) OR (Kasai Procedure[Title/Abstract])) OR (Hepatic Portoenterostomy[Title/Abstract])) OR (Hepatic Portoenterostomies[Title/Abstract])) OR (Portoenterostomies, Hepatic[Title/Abstract])) AND ((((((((((Biliary Atresia[MeSH Terms]) OR (Atresia, Biliary[Title/Abstract])) OR (Intrahepatic Biliary Atresia[Title/Abstract])) OR (Atresia, Intrahepatic Biliary[Title/Abstract])) OR (Biliary Atresia, Intrahepatic[Title/Abstract])) OR (Biliary Atresia, Extrahepatic[Title/Abstract])) OR (Atresia, Extrahepatic Biliary[Title/Abstract])) OR (Extrahepatic Biliary Atresia[Title/Abstract])) OR (Idiopathic Extrahepatic Biliary Atresia[Title/Abstract])) OR (Familial Extrahepatic Biliary Atresia[Title/Abstract])) |

## Embase:

| #1 | 'portoenterostomy'/exp OR 'portoenterostomy' OR 'hepatic portoenterostomy':ab,kw,ti OR 'hepato-porto-enterostomy':ab,kw,ti OR 'hepato-portoenterostomy':ab,kw,ti OR 'hepatoportoenterostomy':ab,kw,ti OR 'kasai operation':ab,kw,ti OR 'kasai porto-enterostomy':ab,kw,ti OR 'kasai portoenterostomy':ab,kw,ti OR 'kasai procedure':ab,kw,ti OR 'portoenterostomy, hepatic':ab,kw,ti OR 'portointestinal shunt':ab,kw,ti OR 'portoenterostomy':ab,kw,ti |
| --- | --- |
| #2 | 'bile duct atresia'/exp OR 'atresia, bile duct':ab,kw,ti OR 'bile duct agenesia':ab,kw,ti OR 'bile duct agenesis':ab,kw,ti OR 'biliary atresia':ab,kw,ti OR 'biliary duct agenesis':ab,kw,ti OR 'biliary duct atresia':ab,kw,ti OR 'biliary tract atresia':ab,kw,ti OR 'common bile duct agenesis':ab,kw,ti OR 'congenital bile duct atresia':ab,kw,ti OR 'congenital biliary atresia':ab,kw,ti OR 'extrahepatic biliary atresia':ab,kw,ti OR 'bile duct atresia':ab,kw,ti |
| #3 | #1 AND #2 |

## Cochrane library:

| #1 | MeSH descriptor: [Portoenterostomy, Hepatic] explode all trees |
| --- | --- |
| #2 | (Hepatic Portoenterostomies):ti,ab,kw OR (Hepatic Portoenterostomy):ti,ab,kw OR (Hepatoportoenterostomy):ti,ab,kw OR (Portoenterostomies, Hepatic):ti,ab,kw OR (Kasai Procedure):ti,ab,kw OR (Hepatoportoenterostomies):ti,ab,kw |
| #3 | #1 OR #2 |
| #4 | MeSH descriptor: [Portoenterostomy, Hepatic] explode all trees |
| #5 | (Extrahepatic Biliary Atresia):ti,ab,kw OR (Biliary Atresia, Extrahepatic):ti,ab,kw OR (Atresia, Extrahepatic Biliary):ti,ab,kw OR (Idiopathic Extrahepatic Biliary Atresia):ti,ab,kw OR (Familial Extrahepatic Biliary Atresia):ti,ab,kw OR (Biliary Atresia, Intrahepatic):ti,ab,kw OR (Intrahepatic Biliary Atresia):ti,ab,kw OR (Atresia, Intrahepatic Biliary):ti,ab,kw OR (Atresia, Biliary):ti,ab,kw |
| #6 | #4 OR #5 |
| #7 | #3 AND #6 |
